# New hybrid sequencing data of *Vreelandella sulfidaeris* DSM 15722^T^ and *Vreelandella aquamarina* DSM 15723 provide circular and complete genomes

**DOI:** 10.1016/j.dib.2026.112707

**Published:** 2026-03-19

**Authors:** Gwendoline Marquet, Margareth Renault, Françoise Irlinger, Vincent Hervé

**Affiliations:** Université Paris-Saclay, INRAE, AgroParisTech, UMR SayFood, 91120 Palaiseau, France

**Keywords:** *Halomonadaceae*, *Halomonas*, Halophilic bacteria, Resequencing, PacBio, Illumina, Genomics

## Abstract

*Vreelandella* is a genus of halophilic and Gram-negative bacteria belonging to the family *Halomonadaceae* (*Pseudomonadota*). Various genomes of this genus are currently available but their quality is uneven. Hybrid sequencing is now a common practice for generating complete and high-quality circular genomes. Combining long-read (PacBio) and short-read (Illumina) sequencing data, we report here the circular and complete genomes of the type strain *Vreelandella sulfidaeris* DSM 15722^T^ (4.5 Mbp with 53.7% G + C content) and of the strain *Vreelandella aquamarina* DSM 15723 (3.6 Mbp with 56.8% G + C content). The resequencing of these genomes provides an updated and high-quality dataset that can be used to improve our understanding of the metabolism of the *Vreelandella* genus. These genomes could be easily reused to address broader ecological and evolutionary questions related to this genus.

Specifications Table

**Table:** 

Subject	Biology
Specific subject area	Genomics, bacteriology
Type of data	Table Genomic data, Raw, Processed
Data collection	Genomic DNA extraction: NucleoMag HMW DNA kit (Macherey-Nagel) Genome sequencing platforms: Illumina Nova Seq X PE150 platform and PacBio Revio platform
Data source location	The bacterial strains were obtained from the German Collection of Microorganisms and Cell Cultures (DSMZ). The data were collected and stored at the SayFood Unit, Laboratory of INRAE, Palaiseau, France.
Data accessibility	-Repository name: NCBI (https://www.ncbi.nlm.nih.gov/) Data identification number: Bioprojects PRJNA1377305 and PRJNA1381297 Direct URL to data: DSM 15722^T^: https://www.ncbi.nlm.nih.gov/bioproject/PRJNA1377305 https://www.ncbi.nlm.nih.gov/biosample/SAMN53768409 https://www.ncbi.nlm.nih.gov/nuccore/JBSVSF000000000.1 DSM 15723: https://www.ncbi.nlm.nih.gov/bioproject/PRJNA1381297 https://www.ncbi.nlm.nih.gov/biosample/SAMN54104122 https://www.ncbi.nlm.nih.gov/nuccore/JBSXYF000000000.1 Instructions for accessing these data: The complete genome sequences of *Vreelandella sulfidaeris* DSM 15722^T^ and *Vreelandella aquamarina* DSM 15723 are available in the National Center for Biotechnology Information (NCBI) database. -Repository name: Recherche Data Gouv (https://recherche.data.gouv.fr/en) Direct URL to data: https://entrepot.recherche.data.gouv.fr/dataverse/vreelandella_genome_resequencing Instructions for accessing these data: All the annotations described in this paper are freely available from this repository.
Related research article	None

## 1 Value of the Data

• This study presents a complete and circular genome of *Vreelandella aquamarina* DSM 15723 and *Vreelandella sulfidaeris* DSM 15722^T^.
• This dataset provides higher-quality sequencing data compared to the currently available genomes of these strains.
• This dataset is a valuable resource for comparative genomics.
• This dataset may facilitate research into the metabolism, ecology and evolution of the *Vreelandella* genus.

## 2 Background

*Vreelandella* is a genus of Gram-negative bacteria belonging to the halophilic family *Halomonadaceae*. The overall taxonomy of this large and diverse family has been recently revised based on genomic data [1]*.* Indeed, bacterial genomes are now routinely used in taxonomy and systematics to infer phylogenetic trees [2] and for comparative genomics [3]. Therefore, high-quality and complete genomes are needed for accurate and relevant genomic analyses.

At the time of writing, 41 species of *Vreelandella* have been validly published under the International Code of Nomenclature of Prokaryotes (ICNP). Currently many *Vreelandella* genomes are available but their quality is sometimes uneven [1]. Among them, the draft genomes of *Vreelandella sulfidaeris* DSM 15722^T^ (type strain) and *Vreelandella aquamarina* (synonym *Halomonas axialensis*) DSM 15723, two strains isolated from deep-sea hydrothermal-vent environments [4], have been published [5,6]. Here we report the circular and complete genomes of *Vreelandella sulfidaeris* DSM 15722^T^ (type strain) and *Vreelandella aquamarina* DSM 15723. The resequencing of these genomes provides an updated dataset that can be used to better understand the metabolism, ecology and evolution of the *Vreelandella* genus.

## 3 Data Description

We present the complete and circular genomes of *Vreelandella aquamarina* DSM 15723 and *Vreelandella sulfidaeris* DSM 15722^T^, obtained through hybrid sequencing that combines Illumina NovaSeq X PE150 and PacBio Revio data. We compared our data with previously published genomes generated by Oxford Nanopore Technology (ONT) (Table 1).

**Table 1:** Genome assembly and annotation characteristics of the two *Vreelandella* strains, obtained via ONT and hybrid sequencing methods.

	*Vreelandella sulfidaeris* DSM 15722^T^		*Vreelandella aquamarina* DSM 15723	
Sequencing technology	ONT	PacBio/Illumina	ONT	PacBio/Illumina
Genome size (bp)	4,480,770	4,504,718	3,619,799	3,629,439
Coverage (x)	98	72	100	70
Nb of contigs	2	2	1	2
GC %	53.7	53.7	56.8	56.8
CDS	7,620	4,212	5,603	3,365
Coding density (%)	82	89	85.8	90.2
Pseudogenes	119	17	1,019	8
Hypothetical genes	1,804	334	1,735	154
rRNA	18	18	18	18
tRNA	62	62	62	62
tmRNA	1	1	1	1
ncRNA	5	6	11	12
CheckM2 completeness (%)	82.8	100	84.76	99.99
CheckM2 contamination (%)	6.56	1.08	4.99	0.42
GenBank genome assembly	GCA_007182875.1	GCA_054072535.1	GCA_007163885.2	GCA_054110395.1

Concerning the strain *V. sulfidaeris* DSM 15722^T^, the two different sequencing resulted in two contigs (1 chromosome and 1 plasmid), with a similar total genome size. However, for the strain *V. aquamarina* DSM 15723, while one contig was detected using Nanopore technology, the analysis of hybrid sequencing revealed the presence of two contigs (1 chromosome and 1 plasmid).

Regarding the coding density, we found higher values than for ONT sequenced genomes. Conversely, the number of pseudogenes (17 *vs* 119 for DSM 15722^T^ and 8 *vs* 1,019 for DSM 15723), hypothetical genes (1,804 *vs* 334 for DSM 15722^T^ and 1,735 *vs* 154 for DSM 15723) and coding DNA sequence (CDS) (7,620 *vs* 4,212 for DSM 15722^T^ and 5,603 *vs* 3,365 for DSM 15723) are lower in our current dataset. Overall, our dataset offers genomes of higher quality with significantly higher completeness and lower contamination as determined by CheckM2 criteria (Table 1).

We also examined the predicted secondary metabolism using antiSMASH and found more regions encoding biosynthetic gene clusters in our new dataset. For *V. sulfidaeris* DSM 15722^T^, 9 secondary metabolite regions were detected in the ONT sequenced genome. In our newly sequenced genomes, we also detected these nine regions, as well as an extra region related to homoserine lactone production. Similarly, for the strain DSM 15723, five secondary metabolite regions were detected in the ONT sequenced genome. In our complete and circular genome, these five regions were also detected, but we also found two more secondary metabolite regions: one related to the synthesis of a type I polyketide synthase and another related to a cluster of ribosomally synthesized and posttranslationally modified peptide (RiPPs) recognition element (RRE).

## 4 Experimental Design, Materials and Methods

### 4.1 Bacterial culture

The bacterial strains *Vreelandella sulfidaeris* DSM 15722^T^ (= Esulfide1 = ATCC BAA-803 = CECT 5817 = NZp) and *Vreelandella aquamarina* DSM 15723 (= 1723a = Althf1 = ATCC BAA-802 = CECT 5812 = KCTC 22,194) were grown in Marine broth without agitation for 3 days at 28°C. The cell pellets were collected by centrifugation of 1 mL of culture at 5000 x *g* for 10 min at 4°C.

### 4.2 DNA extraction and library preparation

We extracted total DNA using the NucleoMag HMW DNA kit (Macherey-Nagel, Hoerdt, France) following the manufacturer’s protocol with some modifications: we added lysozyme (25 µL, 100 mg/mL) to the bacterial pellet in HM1 buffer, and incubated samples for 1 h at 37°C before the addition of proteinase K (included in the kit). After RNase treatment, samples were mixed with beads. After several steps of washing, the beads were air-dried for 15 min at room temperature. We eluted DNA in 100 µL of 5 mM Tris/HCl, pH 8.5 buffer. DNA quantity and quality were assessed using the Nanodrop One (ThermoFisher Scientific, Waltham, USA) and the Agilent 4150 Tapestation with the Genomic DNA ScreenTape System (Agilent, Santa Clara, USA). Library preparation and sequencing were performed by BMKGENE (Münster, Germany) using two platforms: Illumina NovaSeq X PE150 and PacBio Revio.

### 4.3 Genome assembly and annotation

Previously published genomes of *V. sulfidaeris* DSM 15722^T^ [5] and *V. aquamarina* (previously known as *V. axialensis*) DSM 15723 [6] sequenced using Oxford Nanopore Technology (ONT) were downloaded from NCBI. To compare these two genomes with our newly sequenced ones, we analysed them with the same software with default parameters.

Our genome assembly was performed by Hifiasm v0.12 [7]. Circlator v1.5.5 was applied to circularize the assembled sequences and resolve high-quality ends [8]. The sequence was further corrected using Illumina reads with Pilon v1.22 [9] in order to generate a highly accurate assembly for downstream analysis. Genome annotation was done with Bakta version 1.11.0 [10]. Genome completeness and contamination were assessed by CheckM2 version 1.1.0 [11]. Analysis of secondary metabolism was performed using antiSMASH version 8.0.4 with default parameters [12].

## Limitations

None.

## Ethics Statement

The authors have read and followed the ethical requirements for publication in *Data in Brief.* The current work does not involve human subjects, animal experiments, or any data collected from social media platforms.

## CRediT authorship contribution statement

**Gwendoline Marquet:** Methodology, Validation, Formal analysis, Investigation, Data curation, Writing – original draft, Visualization. **Margareth Renault:** Investigation, Resources, Writing – review & editing. **Françoise Irlinger:** Resources, Writing – review & editing. **Vincent Hervé:** Conceptualization, Validation, Data curation, Writing – original draft, Project administration.

## Data Availability

(NCBI).Genome of Vreelandella sulfidaeris DSM 15722 (Original data)
(NCBI).Genome of Vreelandella aquamarina DSM 15723 (Original data)
